# Zebrafish as a Model to Evaluate a CRISPR/Cas9-Based Exon Excision Approach as a Future Treatment Option for *EYS*-Associated Retinitis Pigmentosa

**DOI:** 10.3390/ijms22179154

**Published:** 2021-08-25

**Authors:** Renske Schellens, Erik de Vrieze, Pam Graave, Sanne Broekman, Kerstin Nagel-Wolfrum, Theo Peters, Hannie Kremer, Rob W. J. Collin, Erwin van Wijk

**Affiliations:** 1 Department of Otorhinolaryngology, Radboud University Medical Center, 6525 GA Nijmegen, The Netherlands; renske.schellens@radboudumc.nl (R.S.); erik.devrieze@radboudumc.nl (E.d.V.); sanne.broekman@radboudumc.nl (S.B.); theo.peters@radboudumc.nl (T.P.); Hannie.Kremer@radboudumc.nl (H.K.); 2 Donders Institute for Brain Cognition and Behaviour, 6500 GL Nijmegen, The Netherlands; rob.collin@radboudumc.nl; 3 Department of Human Genetics, Radboud University Medical Center, 6525 GA Nijmegen, The Netherlands; p.graave@student.ru.nl; 4 Institute for Molecular Physiology, Johannes Gutenberg-University Mainz, 55099 Mainz, Germany; nagelwol@uni-mainz.de; 5 Institute for Developmental Biology and Neurobiology, Johannes Gutenberg-University Mainz, 55099 Mainz, Germany

**Keywords:** *EYS*, CRISPR/Cas9, antisense oligonucleotides, exon skipping therapy, photoreceptors, retinitis pigmentosa, zebrafish

## Abstract

Retinitis pigmentosa (RP) is an inherited retinal disease (IRD) with an overall prevalence of 1 in 4000 individuals. Mutations in *EYS* (*Eyes shut homolog*) are among the most frequent causes of non-syndromic autosomal recessively inherited RP and act via a loss-of-function mechanism. In light of the recent successes for other IRDs, we investigated the therapeutic potential of exon skipping for *EYS*-associated RP. CRISPR/Cas9 was employed to generate zebrafish from which the region encompassing the orthologous exons 37-41 of human *EYS* (*eys* exons 40-44) was excised from the genome. The excision of these exons was predicted to maintain the open reading frame and to result in the removal of exactly one Laminin G and two EGF domains. Although the *eys^Δexon40-44^* transcript was found at levels comparable to wild-type *eys,* and no unwanted off-target modifications were identified within the *eys* coding sequence after single-molecule sequencing, *Eys^Δexon40-44^* protein expression could not be detected. Visual motor response experiments revealed that *eys^Δexon40-44^* larvae were visually impaired and histological analysis revealed a progressive degeneration of the retinal outer nuclear layer in these zebrafish. Altogether, the data obtained in our zebrafish model currently provide no indications for the skipping of *EYS* exons 37-41 as an effective future treatment strategy for *EYS*-associated RP.

## 1. Introduction

Retinitis pigmentosa (RP) is an inherited retinal disease (IRD) with an overall prevalence of 1 in 4000 individuals, affecting almost 2 million individuals worldwide [1,2]. Patients present with a progressive loss of visual function caused by the degeneration of the light-sensitive photoreceptor cells in the retina, often resulting in total blindness in the fifth or sixth decade of life. RP typically manifests with night blindness and visual field constriction from early adolescence onwards, followed by a gradual decrease of visual acuity later in life. This non-congenital onset and slowly progressive nature of RP provides ample time for therapeutic intervention.

To date, mutations in more than 100 genes have been described to cause RP (https://sph.uth.edu/Retnet/; 29 July 2021). Of all RP cases, approximately 60% display an autosomal recessive mode of inheritance (arRP) [3]. Mutations in *Eyes shut homolog* (*EYS*) are a major contributor to arRP pathology, accounting for approximately 5–35% of all cases [4,5,6,7,8,9], and are believed to act via a loss-of-function mechanism.

**Figure 1:**
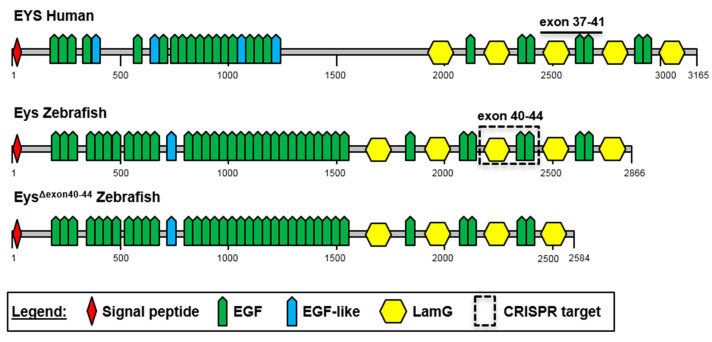
Schematic representation of the domain architecture of human and zebrafish EYS proteins. Both human and zebrafish EYS proteins are comprised of a repetitive protein domain architecture that includes epidermal growth factor (EGF) domains, EGF-like domains and laminin G (LamG) domains. Skipping of eys exon 40-44 results in the exclusion of one LamG and two EGF domains, indicated with a dashed boxed. Numbers indicate amino acids.

*EYS* is located on chromosome 6p12, spans over 2 Mb and is predominantly expressed in the photoreceptor cells of the retina. The full-length transcript is comprised of 44 exons which together encode the 3144 amino acids long protein eyes shut homolog (EYS). The EYS protein domain architecture is predicted to contain 28 epidermal growth factor (EGF)(-like) domains and five laminin G (LamG) domains [10,11] (Figure 1). So far, little is known about the exact function of EYS and the molecular pathogenesis of the associated RP. The EYS protein localizes to the photoreceptor ciliary axonemes and the outer plexiform and ganglion cell layers [12]. However, based on the predicted protein domain architecture, EYS appears to be an extracellular protein.

Currently, no treatment for *EYS*-associated RP exists that can prevent disease progression or restore visual function. For therapy development, suitable cellular or animal models that mimic the human phenotype are pivotal. With the recent developments in induced pluripotent stem cell (iPSC) technology, it is now possible to reprogram iPSCs from easily accessible patient cells (e.g., fibroblasts or PBMCs) and differentiate them into iPSC-derived photoreceptor cells (iPSC-Ps) [13]. This allows the assessment of the molecular efficacy of novel therapeutic strategies in the context of a patient’s own relevant molecular and cellular environment. However, to evaluate the effect of potential therapies on visual function, animal models are essential. Rodents, which are frequently used models for studying IRDs [14], lost the *Eys* gene during evolution [11]. The zebrafish has emerged as an alternative and pre-eminent model to study IRDs, as they have a high fecundity, are amenable to genetic manipulation, and have a retinal structure comparable to humans [14]. Previously, two *eys* knock-out zebrafish models have been generated and characterized [15,16]. The absence of Eys resulted in the loss of photoreceptor structural integrity and an impaired retinal architecture, ultimately leading to mislocalisation of rhodopsin, a progressive degeneration of the photoreceptor cells, and decreased ERG responses. We generated and characterized a third zebrafish knock-out model which, in addition to the previously described phenotypic features, showed diminished locomotor activity in response to light stimuli [17]. Therefore, zebrafish can be regarded as an attractive model to evaluate the potential of novel therapeutic strategies for *EYS*-associated RP.

The development of gene augmentation therapy for *EYS*-associated RP is severely hampered by the size of the EYS-encoding sequence (9.4 kb). The protein-coding sequence exceeds the 4.7 kb packaging capacity of adeno-associated virus (AAV) vehicles, which are the currently preferred vehicles for retinal gene delivery [18]. An alternative therapeutic approach, which has already proven its potential in several (pre-)clinical studies for Usher syndrome, Duchenne muscular dystrophy, and CADASIL [3,19,20], is the antisense oligonucleotide (AON)-based skipping of native, in-frame exons harboring recurrent loss-of-function mutations. In this study, we therefore investigated the therapeutic potential of exon skipping as a future treatment option for *EYS*-associated RP. In order to study the long-term effect of this treatment modality in a controlled setting, we employed the CRISPR/Cas9 system to generate stable zebrafish mutants from which the genomic region that encompasses the orthologous exons of the frequently mutated human *EYS* exons 37-41 was specifically excised. Excision of this in-frame combination of exons, resulting in the stable *eys^Δ^^exon40-44^* zebrafish line, was predicted to result in a transcript encoding a shortened Eys protein that lacks exactly one evolutionary conserved repetitive module consisting of one LamG and two EGF domains. We hypothesized that this protein has sufficient residual function for maintaining vision.

## 2. Results

### 2.1. The C-Terminal Region of EYS Is Highly Conserved between Zebrafish and Man

We, and others, have previously generated and characterized zebrafish *eys* knock-out mutants, and presented the zebrafish as a suitable model to study *EYS*-associated retinal disease [15,16,17]. Our mutant, designated *eys^KO^*, displayed significantly reduced electroretinogram (ERG) traces in the absence of the Eys protein [17]. To identify a potential target region to assess the therapeutic potential of exon skipping as a future treatment option, the conservation of the *EYS* exon structure and protein domain architecture between human and zebrafish was analyzed (Figure 1). We previously reported an overall 33% amino acid identity between human and zebrafish EYS [17]. Zebrafish Eys lacks the so-called low-complexity central region, while the N-terminal EGF domains and EGF-like domains are conserved. The EYS C-terminal region, starting directly after the low-complexity region, and harboring repetitive modules of LamG and EGF domains, shows the highest degree of amino acid conservation (49% identity).

Based on EYS amino acid conservation and the presence of multiple protein-truncating mutations in RP patients, we selected exons 37-41 of human *EYS* as a target to assess the therapeutic potential of exon skipping. *EYS* exons 37-41 encompass 843 nucleotides in the *EYS* mRNA and are predicted to encode exactly one module consisting of one LamG and two EGF domains. In total 18 unique loss-of function mutations have been reported in *EYS* exons 37-41 (*EYS* LOVD mutation database, https://databases.lovd.nl/shared/genes/EYS; 27 July 2021). The length of the orthologous region in the zebrafish *eys* transcript (zebrafish *eys* exons 40-44) is fully conserved, and the encoded protein region shows a 58% amino acid identity. Similar to the human situation, the in-frame deletion of zebrafish *eys* exons 40-44 was predicted to result in a shortened protein (Eys^Δexon40-44^) from which exactly one repetitive module consisting of one LamG and two EGF domains was lost. We hypothesized that this protein has sufficient residual function for maintaining vision.

### 2.2. Eys Is Present at the Periciliary Membrane, Accessory Outer Segment, Connecting Cilium and Ribbon Synapse of Zebrafish Photoreceptor Cells

**Figure 2:**
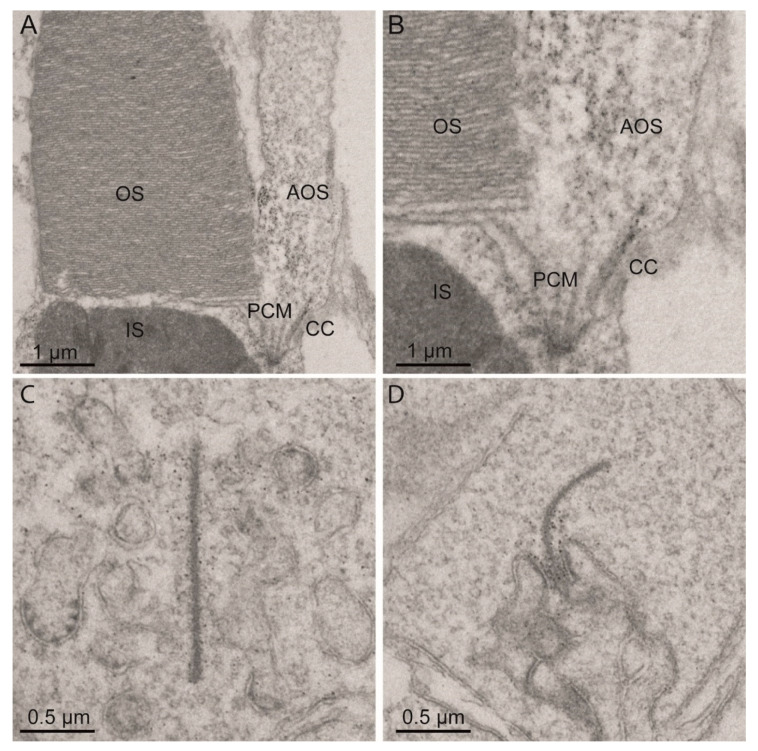
Eys localization in adult zebrafish photoreceptor cells. Immunoelectron microscopy images of adult zebrafish retinas show that Eys localizes at (**A**,**B**) the connecting cilium, periciliary membrane, cone accessory outer segments, and (**C**,**D**) photoreceptor ribbon synapses. CC: connecting cilium, PCM: periciliary membrane, AOS: accessory outer segment, IS: inner segment, OS: outer segment.

In order to determine the exact subcellular localization of Eys in adult zebrafish photoreceptor cells, we performed an immunoelectron microscopic analysis using the previously published anti-Eys antibody [17]. Besides confirming the previously described presence of Eys at the connecting cilium, we also detected Eys at the periciliary membrane, cone accessory outer segment (AOS), and photoreceptor ribbon synapse (Figure 2). AOS are structures that extend from the inner segments of cone photoreceptor cells of teleost fish, run along the outer segments, and are mainly involved in retinomotor movements [21].

### 2.3. Generation of the eys^Δexon40-44^ Zebrafish Line Using CRISPR/Cas9 Technology

**Figure 3:**
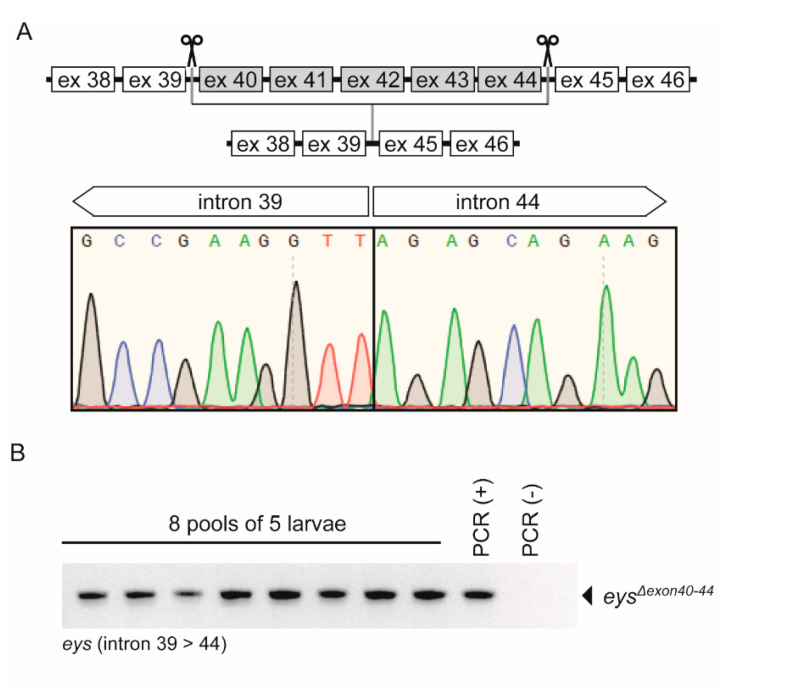
Characterization of the stable *eys^Δexon40-44^* zebrafish line. (**A**) Schematic representation of exon excision approach (upper panel). Sanger sequencing confirmed the presence of the anticipated exons 40-44 excision in injected embryos (1 day post fertilization (dpf)); lower panel). (**B**) PCR analysis identified germline transmission in 8 out of 8 pools of 1 dpf embryos obtained by breeding an injected adult with a wild-type animal. PCR (+): positive PCR control, PCR (-): negative PCR control.

To investigate the therapeutic potential of exon skipping as a future treatment option for *EYS*-associated RP, we adopted the CRISPR/Cas9 technology to generate a stable zebrafish line from which the genomic region encompassing *eys* exons 40-44 was specifically excised. For this, multiple sgRNAs were designed for the genomic regions upstream of *eys* exon 40 and downstream of exon 44, and evaluated for their efficiency to cleave the target DNA by injecting individual sgRNA-Cas9 complexes in fertilized embryos. The most potent sgRNAs upstream of exon 40 and downstream of exon 44 were combined to excise the genomic region in between. To avoid the preferential binding of Cas9 to either sgRNA, individual sgRNA-Cas9 complexes were prepared and mixed together prior to injection. At 1 dpf, 4 out of 16 injected embryos (25%) were screened positive for the anticipated lesion by PCR analysis, and correct excision was confirmed by Sanger sequencing (Figure 3A). The remainder of the injected founder embryos (F0) was raised into adulthood. Forty embryos (8 pools of 5 embryos) of each potential founder were screened for germline transmission by RT-PCR and Sanger sequencing. Germline transmission of the anticipated lesion was identified in the progeny of 1 out of 12 screened F0 founder fish (Figure 3B). To further reduce the incidence of potential off-target editing, the F1 fish harboring the anticipated and sequence verified lesion was outcrossed once more with wild-type TL fish (F2 generation), prior to the generation of the stable homozygous zebrafish *eys* exons 40-44 excision line, designated *eys^Δexon40-44^*. The homozygous *eys^Δexon40-44^* fish were viable and no abnormalities in their overall body morphology, development, or swimming behavior were observed. Finally, PCR analysis and subsequent Sanger sequencing confirmed the absence of any off-target editing events at three predicted off-target sequences with one nucleotide mismatch as compared to the on-target sequence for the sgRNAs used [22] (Figure S1). Due to strain-related differences in the DNA sequence between our zebrafish model (Tupfel Longfin (TL)) and the reference sequence (Tuebingen (TU), assembly: GRCz11/danRer11), one of the three predicted off-target sites had more mismatches with the sgRNA and lacked the PAM-site.

### 2.4. eys^Δexon40-44^ Transcript Analysis

**Figure 4:**
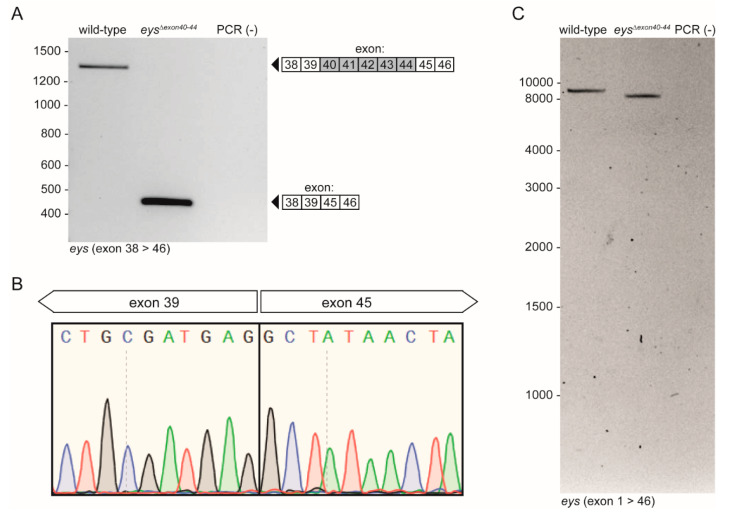
Transcript analysis of *eys^Δexon40-44^* mRNA. (**A**) RT-PCR analysis revealed the absence of *eys* exons 40-44 in *eys^Δexon40-44^* larvae (5 days post fertilization (dpf)), in the absence of any clear alternatively spliced *eys* transcripts. (**B**) Sanger sequencing of the *eys^Δexon40-44^* amplicon confirmed the absence of exons 40-44 from the transcript. (**C**) Long-range PCR analysis did not reveal any additional and unwanted amplicons derived from CRISPR/Cas9-induced alternative splicing of *eys* transcripts. PCR (-): negative PCR control.

Total RNA was isolated from homozygous *eys^Δexon40-44^* zebrafish larvae (*n* = 2) to assess the effect of excision of the genomic region encompassing *eys* exons 40-44 on the transcriptional level. RT-PCR analysis using a forward and reverse primer in respectively exons 38 and 46 of the zebrafish *eys* gene revealed a shortened PCR fragment in the *eys^Δexon40-44^* zebrafish in the absence of any clear alternatively spliced *eys* transcripts (Figure 4A). Sanger sequencing confirmed that the *eys^Δexon40-44^* larvae indeed express the expected *eys* transcript lacking exons 40-44 (Figure 4B). In addition, long-range PCR analysis of the *eys^Δexon40-44^* transcript, followed by PacBio^®^ single-molecule long-read sequencing, did not reveal any aberrant pre-mRNA splicing (Figure 4C) or CRISPR/Cas9-induced off-target modifications within the *eys* coding sequence.

### 2.5. Eys Is Absent from the Retina of 5 dpf eys^Δexon40-44^ Zebrafish

**Figure 5:**
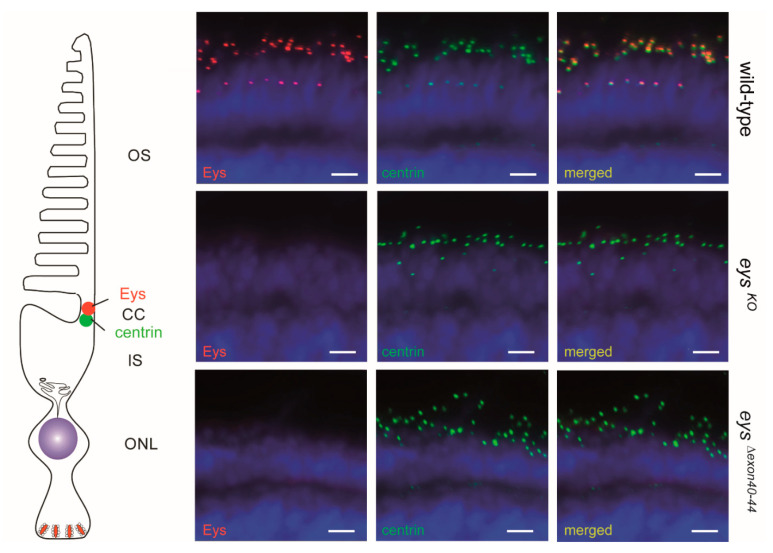
Immunohistochemistry on retinal sections of wild-type, *eys^KO^* and *eys^Δexon40-44^* zebrafish. Retinal cryosections of wild-type, *eys^KO^* and *eys^Δexon40-44^* larvae (5 days post fertilization (dpf)) stained with antibodies directed against Eys (red) and centrin (green). Nuclei are counterstained with DAPI (blue). In *eys^KO^* and in *eys^Δex40-44^* zebrafish retinas no Eys signal was detected, whereas in the retinas of wild-type larvae Eys was present adjacent to the centrin immunoreactivity. Scale bar: 10 μm. OS: outer segment, CC: connecting cilium, IS: inner segment, ONL: outer nuclear layer.

To evaluate whether the excision of zebrafish *eys* exons 40-44 resulted in the generation and correct subcellular localization of a shortened Eys protein (*Eys^Δexon40-44^*) in the retina of homozygous *eys^Δexon40-44^* zebrafish, immunohistochemical analysis was performed using an antibody directed against the N-terminal region of the Eys protein on unfixed retinal cryosections of 5 dpf zebrafish larvae (Figure 5). Eys was previously shown to be present at the region of the photoreceptor connecting cilium, adjacent to the connecting cilium marker centrin [7,17]. As expected, Eys localized adjacent to centrin in strain- and age-matched wild-type larvae, and was absent in the photoreceptors of the *eys^KO^* larvae. Surprisingly, like in the *eys^KO^* zebrafish, no Eys protein could be detected in the retinas of the *eys^Δexon40-44^* larvae, indicating that the excision of exons 40-44 interferes with Eys protein expression in the zebrafish retina. Although our predictions indicate that the epitope to which the anti-Eys antibody is directed is not affected by the excision, it cannot be completely ruled out that the antibody is not able to detect the *Eys^Δexon40-44^* protein.

### 2.6. Disturbed Retinal Morphology and Disorganization of Photoreceptor Outer Segments in Adult eys^Δ^^exon40-44^ Zebrafish

**Figure 6:**
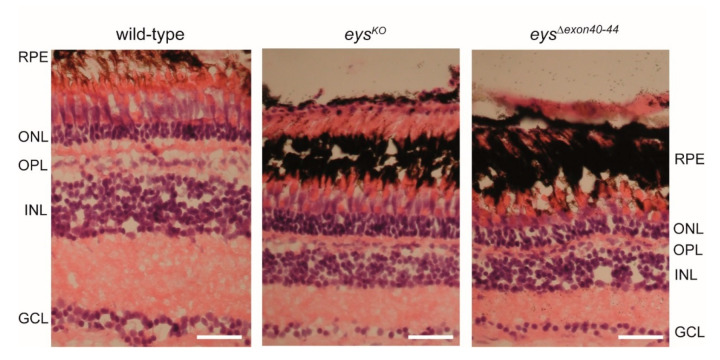
Histological examination of wild-type, *eys^KO^* and *eys^Δexon40-44^* zebrafish retinas. Light microscopy of retinal sections from adult zebrafish (15 months post fertilization (mpf)) stained with hematoxylin (purple) and eosin (red). Retinas of the *eys^Δexon40-44^* and *eys^KO^* lines are morphologically indistinguishable. In both the *eys^Δexon40-44^* and *eys^KO^* retinas, a reduction of the thickness of all retinal layers was observed as compared to wild-type controls. In addition, photoreceptor outer segments seem to be shortened and disorganized in both *eys^Δexon40-44^* and *eys^KO^* as compared to wild-type retinas. Scale bar: 20 μm. RPE: retinal pigment epithelium, ONL: outer nuclear layer, OPL: outer plexiform layer, INL: inner nuclear layer, GCL: ganglion cell layer.

Next, we evaluated whether or not the excision of *eys* exons 40-44 had an effect on the overall retinal morphology. For this, retinal sections of adult zebrafish (15 months post fertilization (mpf)) were stained with hematoxylin and eosin (HE) (Figure 6). Retinas of the *eys^Δexon40-44^* and *eys^KO^* lines appear to be morphologically indistinguishable. In both the *eys^Δexon40-44^* and *eys^KO^* retinas, a reduction in thickness of all retinal layers was observed as compared to strain- and age-matched wild-types. In addition, photoreceptor outer segments were shortened and disorganized in both the *eys^Δexon40-44^* and *eys^KO^* zebrafish as compared to wild-types. These data indicate that the *eys^Δexon40-44^* line is phenotypically highly similar to our previously published *eys^KO^* line [17].

### 2.7. Impaired Locomotor Activity of eys^Δ^^exon40-44^ Zebrafish in Response to Light

**Figure 7:**
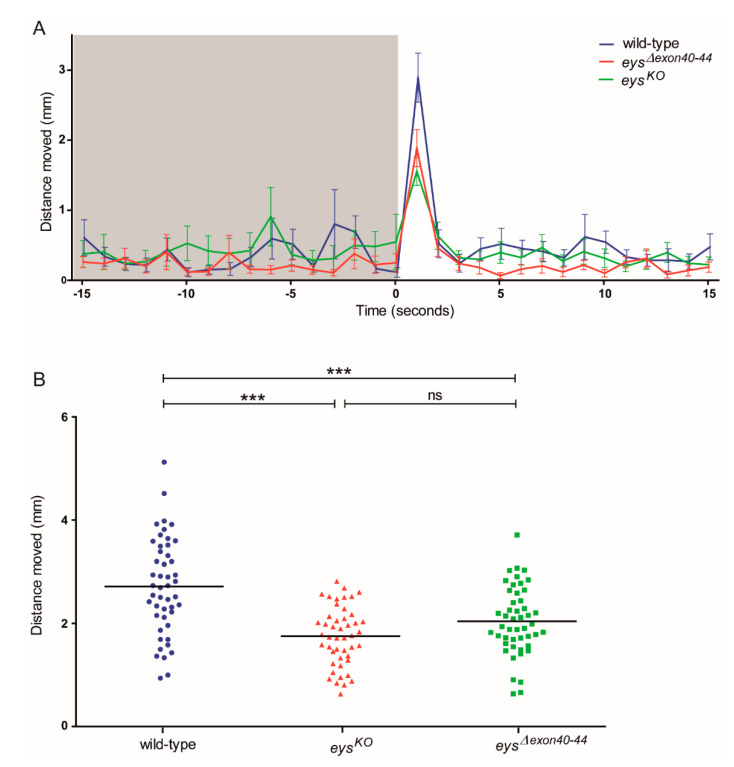
Visual Motor Responses in wild-type, *eys^KO^* and *eys^Δexon40-44^* zebrafish. (**A**) A representative eye-specific Light-ON Visual Motor Response (VMR) from a single trial, presented as the distance moved (mm) per second, is shown for the time frame of 15 s prior to and after light alternation. Note that the response to light is less pronounced in the *eys^KO^* and *eys^Δexon40-44^* larvae as compared to the strain- and age-matched wild-type larvae (5 dpf; *n* = 16 per group). (**B**) The distance moved (mm, mean of 4 Light-ON transitions per larva) during the first second after the Light-ON transition for wild-type, *eys^KO^* and *eys^Δexon40-44^* zebrafish. The distance moved was significantly lower in the *eys^Δexon40-44^* larvae (2.04 mm ± 0.66 (mean ± SD, *n* = 48)) than in wild-type larvae (2.72 mm ± 0.93 (mean ± SD, *n* = 48)), while no significant difference was observed between the *eys^KO^* larvae (1.75 mm ± 0.57 (mean ± SD, *n* = 47)) and *eys^Δexon40-44^* larvae (5 dpf; *n*= 47 larvae). *** indicates *p* < 0.0001. Trials were conducted as 3 biological replicates containing all genotypes in each trial.

Although no sign of *Eys^Δexon40-44^* expression was obtained and the retinal morphology of the adult *eys^Δexon40-44^* zebrafish was severely disturbed, the visual function of the *eys^Δexon40-44^* larvae was assessed using visual motor response (VMR) measurements. The wild-type, *eys^KO^* and *eys^Δexon40-44^* larvae (5 dpf; *n* = 48; 3 biological replicates) were exposed to alternating periods of 50 min of darkness and 10 min of bright light for a total of 5 cycles. We specifically analyzed the distance moved during the first second after the Light-ON transition, since this period is described to contain the visual startle response [23] (Figure 7A). The first technical Light-ON transition is excluded from the analysis since the VMR response to this transition has been described to be altered by the difference in the length of dark adaptation [24]. All larvae analyzed responded to the Light-ON transition, however the response to light was less pronounced in the *eys^KO^* and *eys^Δexon40-44^* larvae as compared to the strain- and age-matched wild-type larvae. Overall, our data showed that the distance moved was significantly lower in the *eys^Δexon40-44^* larvae than in wild-type larvae at two out of four Light-ON transitions (Figure S2; *p* < 0.05 and *p* < 0.001, respectively). If we take the mean distance moved of the four Light-ON transitions per larva, the distance moved was significantly lower in the *eys^Δexon40-44^* larvae than in wild-type larvae (Figure 7B; *p* < 0.0001) Simultaneously, no significant difference in Light-ON VMR was observed between *eys^KO^* and *eys^Δexon40-44^* (Figure 7B, *p* > 0.05; Figure S2, *p* > 0.05), indicating that the excision of *eys* exons 40-44 results in a similar visual function at 5 dpf as observed in the *eys* knock-out model.

## 3. Discussion

Mutations in *EYS* are among the most frequent causes of non-syndromic arRP, accounting for approximately 5–35% of all arRP cases in the European and Asian populations [5,6,7,8,25,26]. In order to investigate the therapeutic potential of exon skipping for the future treatment of *EYS*-associated RP, we generated and characterized a stable zebrafish line from which the region containing the orthologous exons of the frequently mutated human *EYS* exons 37-41, zebrafish *eys* exons 40-44, was specifically excised from the genome using the CRISPR/Cas9 system. The *eys^Δexon40-44^* transcript level was found at levels comparable to that of wild-type *eys* and no off-target modifications within the *eys* coding sequence could be detected by single-molecule long-read transcript sequencing. However, the *Eys^Δexon40-44^* protein could not be detected at the region of the connecting cilium and visual motor response (VMR) experiments revealed that the *eys^Δexon40-44^* larvae were visually impaired. In addition, retinal degeneration was observed in the adult *eys^Δexon40-44^* zebrafish, similar to that in the previously published *eys^KO^* zebrafish. Altogether, the data obtained in our zebrafish model currently provide no indications for the skipping of *EYS* exons 37-41 as an effective future treatment strategy for *EYS*-associated RP.

Since the initial publications in 2008 [10,11], many groups have reported on RP patients carrying mutations in *EYS*. However, limited data are available about the exact function of EYS in the retina and the molecular defects in *EYS*-associated retinal disease. In human Y79 retinoblastoma cells, EYS was identified in the cytoplasm and along the ciliary axoneme [12]. Visualization of EYS in macaque retinal sections revealed EYS proteins in whip-like structures along the photoreceptor ciliary axonemes and in the outer plexiform and ganglion cell layers [12]. As such, EYS was suggested to fulfill a role in the structural organization of the outer segments of mammalian photoreceptors. By performing immunoelectron microscopy, we demonstrated that Eys localizes at the periciliary membrane, cone AOS, connecting cilium and ribbon synapse of zebrafish photoreceptor cells. The exact localization of Eys at the ribbon synapse remains unclear, but may point towards a role in signal transduction. In adult teleost fish, AOS are suggested to provide structural support to the outer segments and are suggested to be involved in the exchange of metabolites between RPE and cones by anchoring the cones to the RPE [21,27,28]. Based on comparisons of ultra-structures of the mammalian and teleost photoreceptors, microtubule structures along the mammalian outer segments were suggested to represent embedded AOS [27]. Our findings are therefore in line with the previously published localization of EYS in whip-like structures along the macaque photoreceptor outer segments and support the hypothesis of EYS to be essential for structural integrity of photoreceptor cells and maintenance of photoreceptor morphology in the mammalian and zebrafish retina [12,15,16,17]. Myosin VIIa and usherin, two other proteins in which mutations have been associated with syndromic forms of RP, have also been shown to localize in the zebrafish cone AOS [21,29]. It is therefore tempting to speculate that these proteins could be involved in the same cellular process, which, upon interruption due to the absence or dysfunction of one of the proteins, might cause RP.

Based on the predicted EYS protein domain architecture, and contradictory to the identified intracellular localization, an additional role for EYS in maintaining structural integrity of the extracellular matrix can be suggested. Usherin and CRB proteins, which are IRD-associated transmembrane proteins, have a somewhat similar protein domain structure compared to EYS, and harbor several EGF(-like) domains and LamG domains [29,30]. Usherin is part of a protein complex that provides protein support to the photoreceptor periciliary region [31]. CRB proteins are known to be involved in photoreceptor morphogenesis and mechanisms that control cell adhesion, polarity, and intracellular communication [30]. This is in line with the previously identified functions of the individual EGF and LamG domains [32,33]. In general, EGF domains and LamG domains are mainly found in the extracellular parts of membrane-bound proteins or in proteins that are secreted, and are amongst others involved in cell signaling and adhesion. In usherin, CRB1, CRB2, and CRB3 the EGF and LamG domains are indeed present in their extracellular regions [29,30]. In contrast, EYS does not contain a transmembrane domain, but at the N-terminal region of the EYS protein a signal peptide is predicted to be present, which might indicate that the protein is secreted and, as such, involved in extracellular processes. It can be argued that the identified intracellular Eys localization captures the translocation of Eys to the extracellular space. Functional studies, for instance protein affinity chromatography and yeast two hybrid assays, might enhance our insights in the function of EYS.

As EYS is thought to be essential for photoreceptor maintenance in humans, therapeutic strategies that rescue the expression of functional EYS protein in the retina can potentially preserve visual function in patients by preventing or slowing down the progression of photoreceptor degeneration. The eye is an easily accessible and immune-privileged organ, which makes it suitable for therapeutic interventions. As such, several genetic therapies for IRDs have reached the clinical phase or even obtained market approval. Patients with *RPE65*-associated RP can now be treated with the first commercially available gene augmentation therapy LUXTURNA™ (voretigene neparvovec-rzyl; Spark Therapeutics, Inc., Philadelphia, PA, USA), which delivers a healthy copy of the *RPE65* cDNA to the retinal cells using an adeno-associated virus (AAV) [34,35]. Antisense oligonucleotides (AON)-induced splice modulation therapies for several IRDs are also in the trajectory to reach the market phase [36]. An advantage of the use of AONs over gene augmentation therapy is that AONs can be more easily delivered. Their small sequence fits in an AAV vector, enabling small nuclear RNA (snRNA)-based AON delivery [37]. snRNAs are components of the small nuclear ribonucleoprotein complex (snRNP), which is involved in histone pre-mRNA processing. The viral delivery of snRNAs with altered, target-specific antisense sequences is now investigated as a versatile, long-term effective splice modulation tool (#NCT04240315). Next to vector-based delivery, splice-modulation AONs can also be delivered as naked molecules. Chemical modifications prevent those naked AONs from RNAse H-based degradation and provide additional benefits to the AONs, such as improved stability, bioavailability and an increased affinity to the target pre-mRNA [38]. AONs target the endogenously expressed pre-mRNA and are therefore not expected to massively in- or decrease expression levels; a potential problem associated with gene augmentation therapies.

Sepofarsen, which prevents the inclusion of a pseudo exon resulting from the frequent c.2991+1655A>G mutation in *CEP290*, has been shown to be effective and safe in a phase 1/2 clinical trial (#NCT03140969) [39] and is currently under investigation in a phase 2/3 trial (#NCT03913143). Besides splice correction, AONs can also be used for the skipping of native exons that harbor disease-causing mutations, provided that the skipping of the respective exons does not affect the reading frame and that the exon does not encode a domain crucial for protein structure or function. AON-mediated exon skipping has already been shown to have a high therapeutic potential for large genes encoding (structural) proteins that are built up by a series of repetitive protein domains, including usherin and dystrophin [3,40]. The safety and therapeutic efficacy of AON-induced skipping of native exons has been explored most thoroughly for Duchenne muscular dystrophy (caused by mutations in the *DMD* gene), and two AON-based treatments have recently obtained market approval by the FDA [41,42,43,44,45]. In the same way, QR-421a-based skipping of *USH2A* exon 13 (642 bp) has already been successfully tested in a phase 1/2 clinical trial (#NCT03780257) and two pivotal phase 2/3 clinical trials are currently being planned [3]. In contrast to the previously mentioned *DMD-* and *USH2A*-related therapeutic exon skipping strategies, the *EYS* region that we aimed to skip consists of multiple exons. In a study by Aartsma-Rus et al. [46], AON-mediated co-skipping of *DMD* exon 43 and 44 resulted in the restoration of dystrophin protein expression levels in patient myotubes, suggesting that a double exon skipping approach is feasible. However, it remains to be determined whether or not it is also possible to induce the simultaneous skipping of five exons.

Different from AON-induced exon skipping strategies, a CRISPR/Cas9-mediated exon-excision strategy physically removes both the targeted exonic DNA regions and interspaced intronic regions. This approach has recently been suggested by Pendse et al. [47] as a potential treatment for *USH2A*-associated disease caused by mutations in exon 13. In order to study the long-term effect of exon skipping as a future treatment option for *EYS*-associated RP, we employed the CRISPR/Cas9 system to generate stable zebrafish mutants from which the genomic region that encompasses the orthologous exons 37-41 of human *EYS* (*eys* exons 40-44) was specifically excised. To our surprise, we could not detect any *Eys^Δexon40-4^*^4^ protein in the retina of the *eys^Δexon40-44^* larvae. The *eys^Δexon40-44^* transcript seems to be present at levels comparable to wild-type *eys*, and the removed introns are not located proximal to the promotor [48]. Although introns can influence transcript levels by affecting the rate of transcription, nuclear export, and transcript stability [49], the influence of the removed introns on transcript levels is concluded to be negligible. Next to influencing transcription, introns can also influence the efficiency of mRNA translation. One possible explanation for the lack of *Eys^Δexon40-44^* protein expression in the zebrafish retina could be an altered secondary structure due to the loss of exons 40-44 from the mature transcript. Erdmann-Pham et al. [50] showed translation to be a complex process that depends on many parameters, including the traffic flow of ribosomes along the mRNA, sensitivity to initiation rate changes, and efficiency of ribosome usage. The deletion of *eys* exons 40-44 from the transcript might influence translation efficiency, for example by blocking ribosomes from binding to the RNA due to an altered secondary structure, therewith limiting translation initiation. In addition, the altered secondary structure might hamper the traffic flow of the ribosomes along the mRNA, thus attenuating translation elongation. Another possible explanation for the perceived absence of the *Eys^Δexon40-44^* protein in the zebrafish retina is the potential change in folding of the protein that interferes with the ability of the antibody to detect the Eys^Δexon40-44^ protein. Although our predictions indicate that the epitope to which the anti-Eys antibody is directed is not affected by the excision, it cannot be completely ruled out that the antibody is not able to detect the *Eys^Δexon40-44^* protein.

We previously performed a multiple sequence alignment using the EYS amino acid sequences from different species and observed a high degree of conservation of the EYS sequence and protein domain architecture [17]. Based on EYS amino acid conservation and the presence of multiple protein-truncating mutations in RP patients [51], we selected human *EYS* exons 37-41 as a target to assess the therapeutic potential of exon skipping. However, an in-depth study on evolutionary conservation might provide more insight in to which regions are essential for EYS protein expression and/or function. Perhaps the regions that are poorly conserved or even absent in other species, such as zebrafish and drosophila, are key to the function of EYS in the human photoreceptors. Alternatively, a lack of conservation could indicate protein domains with biological functions that are not selected for during evolution, and can therefore be missed in humans. In addition, long-read sequencing technologies using tissue from healthy individuals might help to identify naturally occurring alternative splicing events and shed light on which in-frame exons can be missed without influencing protein expression or function. Vice versa, pathogenic in-frame deletions or exon skipping events as a consequence of pathogenic variants also indicate which (in-frame) exons are essential for protein expression or function [52].

We conclude that the genomic region that encompasses the orthologous exons of the frequently mutated human *EYS* exons 37-41 is essential for Eys protein expression in the zebrafish retina. The data obtained in our zebrafish model therefore currently provide no indications for skipping of *EYS* exons 37-41 to be a feasible future treatment strategy for *EYS*-associated RP. The zebrafish is a pre-eminent model to study IRDs as they have a high fecundity, are amenable to genetic manipulation, and have a retinal structure comparable to humans [14]. As previously published for *USH2A-*associated retinal degeneration, proof of concept of exon-skipping therapy obtained in zebrafish has high translational value [3]. However, due to inter-species differences in the Eys protein and presence of translation regulatory elements in the deleted intronic regions, the effect of AON-induced skipping of exons 37-41 of human *EYS* on EYS expression and localization could be different. As a next step, the analysis of EYS protein expression after AON- or CRISPR/Cas9-mediated exon skipping in a suitable human cellular model (e.g., iPSC-derived 3D retinal organoids) will determine the translational value of the results obtained from our zebrafish studies and whether or not it is worthwhile to further explore this therapeutic approach using alternative cellular or animal models.

## 4. Materials and Methods

### 4.1. Zebrafish Ethics, Maintenance and Husbandry

Animal experiments were conducted in accordance with the Dutch guidelines for the care and use of laboratory animals (Wet op de Dierproeven 1996) and European regulations (Directive 86/609/EEC), with the approval of the Central Committee Animal Experimentation (Centrale Commissie Dierproeven [CCD]) of the Royal Netherlands Academy of Arts and Sciences (Koninklijke Nederlandse Akademie van Wetenschappen [KNAW]) (Protocol #RU-DEC2016-0091). Wild-type Tupfel Longfin (TL) zebrafish and the previously described *eys^rmc101/rmc101^* mutants, here designated *eys^KO^*, were used [17]. Embryos were obtained from natural spawning. Zebrafish were maintained and raised according to standard methods [53].

### 4.2. Multiple Sequence Alignment

A multiple sequence alignment of the C-terminal part of the human EYS protein (encoded by exons 27-44; NM_001142800.1) and zebrafish Eys protein (encoded by exons 30-46) [17] was generated using AlignX in the Vector NTI software package (Vector NTI Advance 11).

### 4.3. Fixation and Pre-Embedding Labeling for Immunoelectron Microscopy

For immunoelectron microscopy of adult zebrafish retinas, we followed the previously published protocol for pre-embedding labeling [29,54,55]. In brief, rabbit anti-EYS (1:300; Novus Biologicals, Centennial, CO, USA, #NBP1-90038) was applied on vibratome sections of pre-fixed (4% paraformaldehyde) dissected adult zebrafish eyes, followed by incubation with biotinylated secondary antibodies. Antibody reactions were visualized by a Vectastain ABC-Kit (Vector Laboratories, Burlingame, CA, USA) and a 0.01% hydrogen peroxide to 0.05 M diaminobenzidine (DAB) solution was added. Stained retinas were fixed in 2.5% glutaraldehyde in a 0.1 M cacodylate buffer (pH 7.4), followed by silver enhancement of DAB precipitates and post-fixation in cacodylate buffered 0.5% OsO_4_ on ice. Dehydrated specimens were flat-mounted between two sheaths of ACLAR-film (Ted Pella Inc., Redding, CA, USA) in Araldite resin. Ultrathin sections were made using a Reichert Ultracut S ultramicrotome (Leica, Wetzlar, Germany), collected on Formvar-coated copper or nickel grids and counterstained with 2% uranyl acetate in 50% ethanol and aq. 2% lead citrate. Ultrathin sections were analyzed in a Tecnai 12 BioTwin transmission electron microscope (FEI, Eindhoven, The Netherlands). Images were obtained with a CCD camera (charge-coupled-device camera; SIS MegaView3; Surface Imaging Systems, Herzogenrath, Germany) and processed with Adobe Photoshop CS (Adobe Systems).

### 4.4. CRISPR/Cas9 Genome-Editing Design

For genome editing, target sites were selected using the online web tool CHOPCHOP (https://chopchop.cbu.uib.no/; 27 July 2018) [56]. Single guide RNAs (sgRNAs) were selected for synthesis based on the amount of predicted, off-target sites and by the Doench et al. [57] predicted efficiency score. The synthesis of sgRNAs was performed as described previously [58]. In brief, templates for in-vitro sgRNA transcription were generated by annealing target-specific oligonucleotides containing the T7 promoter sequence (5′-TAATACGACTCACTATA-3′), the 20-base target sequence, and a region (5′-GTTTTAGAGCTAGAAATAGCAAG-3′) complementary to a constant oligonucleotide encoding the reverse complement of the tracrRNA tail. Phusion^™^ High-Fidelity DNA Polymerase (New England Biolabs, Ipswich, MA, USA #M0530L) was used to fill the ssDNA overhang, after which the template was purified using the GenElute^™^ PCR clean-up kit (Sigma, Saint Louis, MO, USA, #NA1020-1KT). Transcription of the sgRNAs was performed using the T7 MEGAshortscript^™^ Kit (Thermo Fisher, Waltham, MA, USA, #AM1354). Obtained transcripts were purified using the MEGAclear™ Transcription Clean-Up Kit (Thermo Fisher, Waltham, MA, USA, #AM1908). The oligonucleotides used for sgRNA synthesis are listed in Table S1.

### 4.5. Microinjections

For the generation of the *eys^Δexon40-44^* zebrafish (designated *eys^rmc9^* in ZFIN), the 5′ sgRNA, 3′ sgRNA and commercial Alt-R^®^ S.p. Cas9 Nuclease V3 (IDT, Coralville, IA, USA, #1081059) were co-injected. To avoid preferential in vivo binding of Cas9 to either sgRNA, individual sgRNA-Cas9 complexes were prepared and mixed together prior to injection. For this, the individual mixtures were incubated at 37°C for 5 min, after which they were combined prior to injection. The final injection mix contained 80 ng/μL 3′ sgRNA, 80 ng/μL 5′ sgRNA, 800 ng/μL Cas9 protein, 0.2 M KCl and 0.05% phenol red. Injection needles (World Precision Instruments, Friedberg, Germany, #TW120F-3) were prepared using a micropipette puller (Sutter Instrument Company, Novato, CA, USA, Model P-97). Wild-type zebrafish embryos were collected after natural spawning and injected at the single cell stage with 1 nL of injection mixture using a Pneumatic PicoPump pv280 (World Precision Instruments, Friedberg, Germany). After injection, the embryos were raised at 28.5 °C in an E3 embryo medium (5 mM NaCl, 0.17 mM KCl, 0.33 mM CaCl_2_, and 0.33 mM MgSO_4_), and supplemented with 0.1% (*v*/*v*) methylene blue. At 1 day post fertilization (dpf), part of the injected embryos (8 pools of 5 embryos) was analyzed for the presence of the anticipated exon deletion using genomic PCR analysis. The remainder of the injected embryos were raised to adulthood.

### 4.6. Genotyping

Genomic DNA was extracted from whole larvae (1 dpf) or caudal fin tissue from adult zebrafish. Tissue was lysed in 25 μL (larvae) or 75 μL (fin tissue) lysis buffer (40 mM NaOH, 0.2 mM EDTA) at 95 °C for 20 min. The lysed samples were diluted 10 times with milli-Q water, after which 1 μL of the diluted sample was used as a DNA template in 2 PCR reactions to amplify the wild-type zebrafish *eys* allele and the zebrafish *eys^Δexon40-44^* allele, and in 3 PCR reactions to screen the predicted off-target sequences containing 1 mismatch as compared to the on-target sequence for both sgRNAs used. One nucleotide mismatch off-target sequences is listed in Table S2. For PCR analysis, the Q5 High-Fidelity DNA Polymerase kit (New England Biolabs, Ipswich, MA, USA, #M0491L) was employed. All primer sequences are listed in Table S3. NEB Tm Calculator (https://tmcalculator.neb.com/#!/main, 2 August 2018) was used to obtain primer annealing temperatures (Tm). The cycling conditions were as follows: 98 °C for 2 min, 35 cycles of 98 °C for 10 s, Tm for 20 s and 72 °C for 30 s, followed by a final 72 °C for 2 min. The presence or absence of the *eys^Δexon40-44^* allele and the absence of screened off-target mutations was confirmed by Sanger sequencing.

### 4.7. Transcript Analysis

For *eys^Δexon40-44^* transcript analysis, 2 *eys^Δexon40-44^* or 2 wild-type larvae (5 dpf) were pooled and snap frozen in liquid nitrogen and total RNA was isolated using the RNeasy^®^ Micro kit (Qiagen, Hilden, Germany #74004) according to manufacturer’s protocol. Subsequently, 100 ng of total RNA was used as a template for cDNA synthesis using the SuperScript^™^ IV Reverse Transcriptase kit (Thermo Fisher, Waltham, MA, USA, #18090200). A fragment spanning the deleted exons of the *eys* gene was amplified from the synthesized cDNA using Q5^®^ High-Fidelity DNA Polymerase kit (New England Biolabs, Ipswich, MA, USA #M0491L) and using a forward primer and a reverse primer located in exons 38 and 46 of the zebrafish *eys* gene, respectively. Primer sequences are listed in Table S3. The cycling conditions were as follows: 98 °C for 1 min, 35 cycles of 98 °C for 10 s, 60 °C for 20 s and 72 °C for 30 s, followed by a final 72 °C for 5 min. Amplified fragments were separated on a 1% agarose gel and sequence-verified by Sanger sequencing.

### 4.8. Targeted Transcript Sequencing

To exclude the presence of CRISPR/Cas9-induced off-target edits within the *eys* coding sequence, pools of five *eys^Δexon40-44^* or wild-type larvae (5 dpf) were snap frozen in liquid nitrogen and total RNA was extracted using the NucleoSpin^®^ RNAII Isolation kit (Macherey-Nagel, Hœrdt, France, #740955.50) according to manufacturer’s protocol. Subsequently, 1 µg of total RNA was used as a template for cDNA synthesis using the SuperScript^™^ IV Reverse Transcriptase kit (Thermo Fisher, Waltham, MA, USA, #18090200) and oligo dT primers. To visualize potential CRISPR/Cas9-induced rearrangements, part of the cDNA was amplified using Q5^®^ High-Fidelity DNA Polymerase kit (New England Biolabs, Ipswich, MA, USA #M0491L) and primers targeting the full length *eys* transcript (exons 1-46). Part of the amplified *eys* exons 1-46 fragment was used as an input for a nested PCR to increase the amount of the amplicon. All primer sequences are listed in Table S3. The cycling conditions were as follows: 98 °C for 2 min, 35 cycles of 98 °C for 10 s, 67 °C (full-length amplicon) or 65 °C (nested amplicon) for 20 s and 72 °C for 6 min, followed by a final 72 °C for 5 min. The concentrations of the wild-type and *eys^Δexon40-44^* zebrafish amplicons resulting from the nested PCR were determined using a Qubit fluorometer (Thermo Fisher, Waltham, MA, USA). Subsequently, 500 ng of each amplicon was analyzed by PacBio^®^ single-molecule long-read sequencing. PacBio^®^ SMRTbell barcoded libraries were prepared according to protocol ‘Procedure and Checklist—Preparing SMRTbell Libraries using PacBio Barcoded Adapters for Multiplex SMRT Sequencing’ (Pacific Biosciences, Menlo Park, CA, USA, Part Number 100-538-700-02). SMRTbell barcoded libraries were loaded on a 1 M SMRTcell and sequenced on the PacBio^®^ Sequel I system (Pacific Biosciences, Menlo Park, CA, USA). SMRT^®^ Link version 10.0 software was used for demultiplexing and High Fidelity (HiFi) CCS3 (Circular Consensus Sequencing) read generation and mapping. Variant calling was performed using the JSI SeqNext version 5.1.0 Build 503 software.

### 4.9. Immunohistochemistry and Histology

Zebrafish *eys^Δexon40-44^*, *eys^KO^* and strain-matched wild-type larvae (5 dpf) were cryoprotected with 10% sucrose in PBS for 10 min prior to embedding in an OCT compound (Sakura, Alphen aan den Rijn, The Netherlands, Tissue-Tek, #4583). After embedding, samples were snap frozen in liquid nitrogen-cooled isopentane and cryosectioned following standard protocols. Cryosections (7 μm thickness along the lens/optic nerve axis) were permeabilized for 20 min with 0.01% Tween-20 in PBS. Sections were rinsed 3 times for 5 min with PBS and blocked for 1 h with a blocking buffer (10% normal goat serum and 2% bovine serum albumin in PBS). The antibodies diluted in the blocking buffer were incubated overnight at 4 °C. Secondary antibodies were also diluted in the blocking buffer and incubated together with DAPI (1:8000; D1306; Thermo Fisher, Waltham, MA, USA) for 1 h. Sections were post fixed with 4% paraformaldehyde for 5 min and mounted with Prolong Gold Anti-fade (P36930; Thermo Fisher, Waltham, MA, USA,). The following primary antibodies and dilutions were used: rabbit anti-EYS (1:300; Novus Biologicals, Centennial, CO, USA, #NBP1-90038), mouse anti-centrin (1:500; Millipore, Burlington, MA, USA; #04-1624). Secondary antibodies (Alexa Fluor 568 goat anti-rabbit (Thermo Fisher, Waltham, MA, USA, #A11011) and Alexa Fluor 647 goat anti-mouse (Thermo Fisher, Waltham, MA, USA, #A21237)) were used in a 1:800 dilution. Images were taken using a Zeiss Axio Imager fluorescence microscope equipped with an AxioCam MRC5 camera (Zeiss, Oberkochen, Germany). To asses adult retinal morphology, dissected adult eyes (15 mpf) from homozygous *eys^Δexon40-44^*, *eys^KO^* and strain-matched wild-type controls were fixed overnight at 4 °C using 4% paraformaldehyde, dehydrated in ascending methanol series, transferred to 100% methanol for an overnight incubation, and rehydrated in a descending methanol series to 0.1% PBS-Tween-20. Afterwards, the eyes were cryoprotected with 10% sucrose in 0.1% PBS-Tween-20 for 15 min, followed by 30% sucrose in 0.1% PBS-Tween-20 for 1 h at room temperature. The larvae were then embedded in the OCT compound ((Sakura, Alphen aan den Rijn, The Netherlands, Tissue-Tek, #4583) and frozen in melting isopentane. Cryosections (7 μm thickness along the lens/optic nerve axis) were stained with hematoxylin and eosin and analyzed using a Zeiss Axioskop light microscope (Zeiss, Oberkochen, Germany).

### 4.10. Visual Motor Response Assay (VMR)

Locomotor activity in response to Light-ON transitions, also known as visual motor response (VMR), was analyzed using EthoVision XT 14 software (Noldus Information Technology BV, Wageningen, The Netherlands). Zebrafish larvae (5 dpf) were individually positioned into a 48-wells plate, containing 350 μL of E3 medium per well. The 48-wells plate was placed in the DanioVision™ observation chamber (Noldus Information Technology BV, Wageningen, The Netherlands). After 1 h of dark adaption, the larvae were exposed to 5 cycles of 50 min dark/10 min light (~14 lux). In each run, 16 *eys^Δexon40-44^* larvae, 16 *eys^KO^* larvae, and 16 strain-matched control wild-type larvae were tested. In all experiments, the larvae were subjected to locomotion analyses between 13:00–18:00 in a sound- and temperature-controlled (28 °C) behavioral testing room. The variable of interest was the distance moved (mm) during the first second after the Light-ON transition. The Graphpad Prism software (version 5.03 for Windows, GraphPad Software, San Diego, CA, USA, www.graphpad.com) was employed to generate scatter plots, calculate mean values and SD values, and perform statistical analysis. VMR data were analyzed using one-way ANOVA using the Tukey method. Statistical significance was set at *p* < 0.05.

## Data Availability

Not applicable.

## Supplementary Materials

The following are available online at https://www.mdpi.com/article/10.3390/ijms22179154/s1.
