# Supplementary material for: Zebrafish as a Model to Evaluate a CRISPR/Cas9-Based Exon Excision Approach as a Future Treatment Option for EYS-Associated Retinitis Pigmentosa
Source: Int J Mol Sci. 2021 Aug 25;22(17):9154. doi: 10.3390/ijms22179154 (PMC8431288; doi:10.3390/ijms22179154)
Supplement: Supplementary file 1 [file ijms-22-09154-s001.zip › ijms-1341551-supplementary.pdf]

International Journal of *Molecular Sciences*

MDPI

Supplementary Materials:

Table S1. Oligonucleotide sequences used for sgRNA synthesis.

**Oligo Name**	**Sequence (5′ > 3′)**
**constant oligo**	AAAAGCACCGACTCGGTGCCACTTTTTCAAGTTGATAACGGAC TAGCCTTATTTTAACTTGCTATTTCTAGCTCTAAAAC
**target-specific oligo 5′ *eys*** $^{\Delta exon40-44}$	CCGCTAGC**TAATACGACTCACTATA***GGCAAAACAAGCCAAACC TT*GTTTTAGAGCTAGAAATAGCAAG
**target-specific oligo 3′ *eys*** $^{\Delta exon40-44}$	CCGCTAGC**TAATACGACTCACTATA***GGTCTTCTGCTCTAAAAAG G*GTTTTAGAGCTAGAAATAGCAAG

T7 promoter in bold. Gene specific region in italics. Overlapping regions of the constant and target-specific oligonucleotides are underlined.

Table S2. Predicted off-target loci containing one mismatch compared to the on-target sequence for selected sgRNAs (Assembly: GRCz11/danRer11).

**Location**	**Strand**	**Sequence (5′ > 3′)**
**chr20: 35406954 - 35406976**	-	CCTCCTTTTTAGAGCAGAA**C**ACC
**chr22: 1380801 - 1380823**	+	GGT**G**TTCTGCTCTAAAAAGGAGG
**chr24: 15475502 -15475524**	+	GGT**G**TTCTGCTCTAAAAAGGAGG

Mismatches in bold.

Table S3. Primers used for RT-PCR analysis.

	Primer name	Primer sequence (5′ > 3′)
eys$^{\Delta exon40\text{-}44}$ **genotyping**	*Eys* wild-type or Δexon 40-44 forward	AGGACGATTTTCTGCCTCTG
	Eys Δexon 40-44 reverse	GGGTAAATTCCACATACATATTGAC
	Eys wild-type reverse	GACAGACTAAAACCAGGCCAAA
**CRISPR/Cas9 Off-target genotyping (for off-target sequences containing 1 mismatch compared to on-target sgRNAs)**	chr20: 35406954 – 35406976 forward	TCGACCAGGTTTTGATTGGT
	chr20: 35406954 – 35406976 reverse	GGACTTTCGACCAGGTTTTG
	chr22: 1380801 – 1380823 forward	GTTTCAGCTTGGCGTAGGAG
	chr22: 1380801 – 1380823 reverse	CCGGCTTTGTTTTGGTCTAA
	chr24: 15475502 – 15475524 forward	TGCACCACGTTGTTTACAGG
	chr24: 15475502 – 15475524 reverse	ATAACCCGTTCTGCACTGGA
eys$^{\Delta exon40\text{-}44}$ **transcript analysis**	*Eys* exon 38-47 forward	GGGGCCAAAATATCCAAAAC
	Eys exon 38-47 reverse	AAAGCCAGCAGACAGGGATA
**Long-range PCR**	*Eys* exon 1-20 forward	ATCCTCATCACAGTCCAGGC
	Eys exon 1-20 reverse	AGAACACCTGCAGCAGTTTG
	Eys exon 13-29 forward	TTTTGTGCACCTGGGTTCG
	Eys exon 13-29 reverse	GCTGATGTCTGTGCTTCTCC
	Eys exon 27-46 forward	TGGAGATGAATGAGTGCTGC
	Eys exon 27-46 reverse	AGCTGTTTGGTGTCCTGATAC
	Eys exon 1-46 forward	TCATTGTCTTCCTGCTCAGC
	Eys exon 1-46 reverse	AGCTGTTTGGTGTCCTGATAC
	Eys exon 1-46 forward - nested	AGCCAAAGAACATCAGCGTG
	Eys exon 1-46 reverse - nested	CCAGATGTAACCGATGCCAC

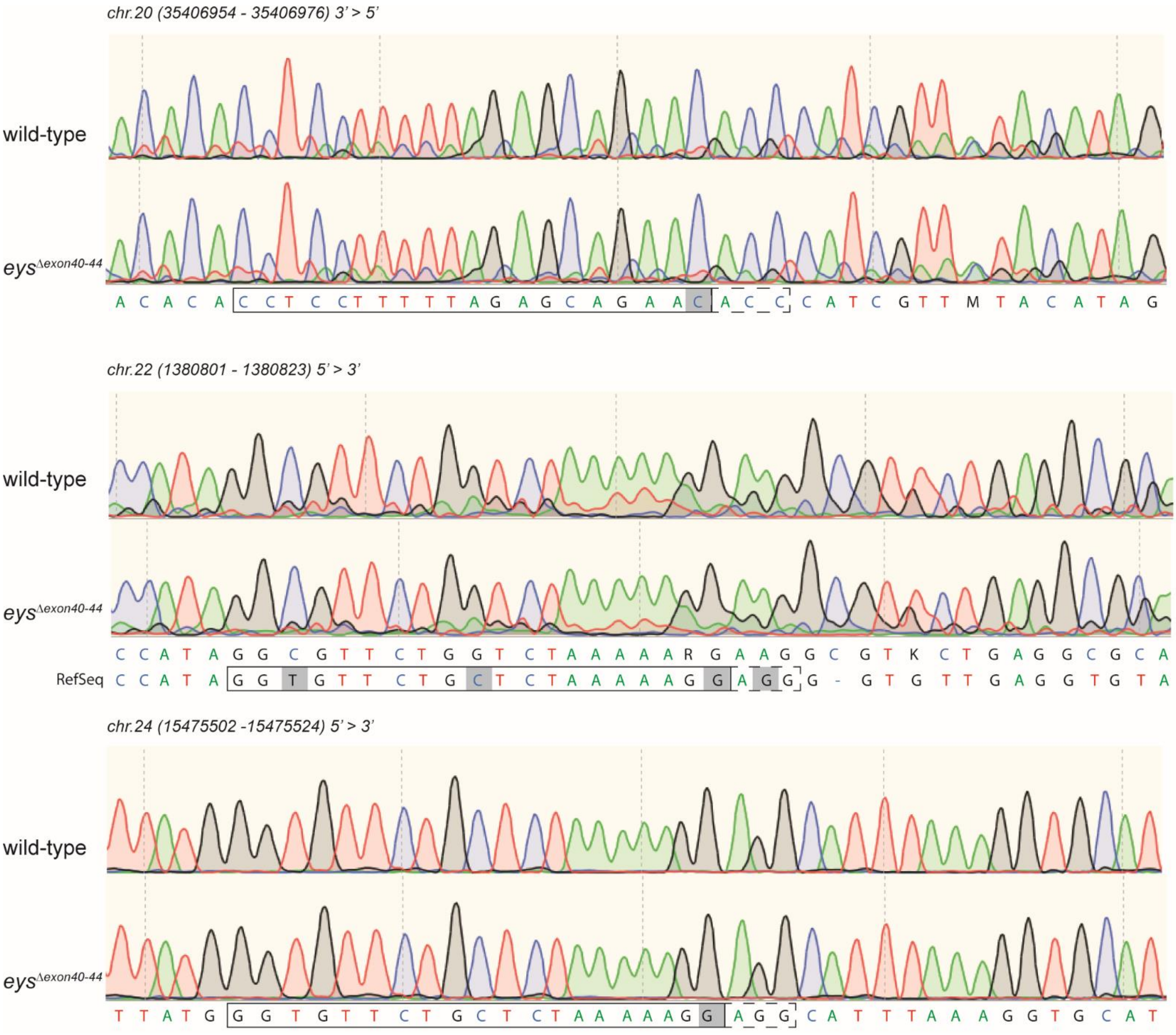

Figure S1. Screening of the predicted off-target loci containing one nucleotide mismatch with the gRNAs used. Sanger sequencing confirmed the absence of any off-target editing events at the three predicted 1-nucleotide mismatch off-target sites. sgRNAs are marked by a box, PAM-sites are marked by a dashed box, mismatches are marked in gray.

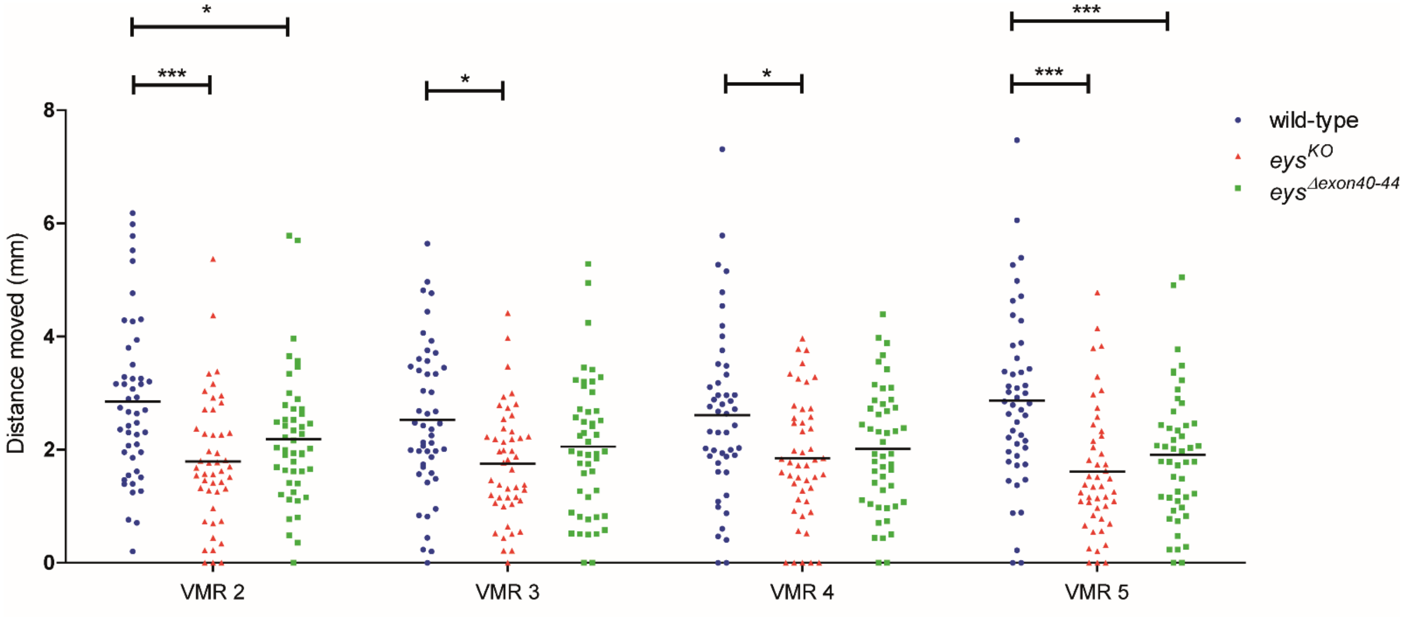

	wild-type			*eys*^{KO}			*eys*$^{\Delta exon40\text{-}44}$		
	Mean	SD	N	Mean	SD	N	Mean	SD	N
VMR 2	2,854	1,412	48	1,795	1,131	47	2,186	1,145	48
VMR 3	2,529	1,313	48	1,751	0,979	47	2,054	1,201	48
VMR 4	2,61	1,507	48	1,85	1,077	47	2,014	1,083	48
VMR 5	2,869	1,521	48	1,615	1,129	47	1,911	1,158	48

Figure S2. Visual Motor Responses in wild-type, *eys*^{KO} and *eys*$^{\Delta exon40\text{-}44}$ zebrafish. The distance moved (mm) during the first second after the Light-ON transition for wild-type, *eys*^{KO} and *eys*$^{\Delta exon40\text{-}44}$ larvae (5 dpf). The distance moved was significantly lower in *eys*$^{\Delta exon40\text{-}44}$ larvae compared to wild-type larvae after two out of four Light-ON transitions, while in none of the transitions a significant difference was observed between *eys*^{KO} and *eys*$^{\Delta exon40\text{-}44}$ larvae (5 dpf; n= 47 larvea, 3 biological replicates, 4 technical replicates). The means and standard deviations (SD) are shown in the table.
